# Parenchymal-Sparing Strategy in Colorectal Liver Metastases: A Single-Center Experience

**DOI:** 10.3390/curroncol33010046

**Published:** 2026-01-15

**Authors:** Eleonora Pozzi, Giuliano La Barba, Fabrizio D’Acapito, Riccardo Turrini, Giulia Elena Cantelli, Giulia Marchetti, Valentina Zucchini, Giorgio Ercolani

**Affiliations:** 1 Department of Medical and Surgical Sciences—DIMEC, Alma Mater Studiorum—University of Bologna, Via Albertoni 15, 40138 Bologna, Italy; eleonora.pozzi3@studio.unibo.it (E.P.); riccardo.turrini2@studio.unibo.it (R.T.); giuliaelena.cantelli@studio.unibo.it (G.E.C.); giulia.marchetti4@studio.unibo.it (G.M.); valentina.zucchini4@studio.unibo.it (V.Z.); giorgio.ercolani@auslromagna.it (G.E.); 2 General and Oncologic Department of Surgery, Morgagni-Pierantoni Hospital, Azienda Unità Sanitaria Locale (AUSL) Romagna, 47121 Forlì, Italy; giuliano.labarba@auslromagna.it

**Keywords:** colorectal liver metastases, parenchymal-sparing hepatectomy, major hepatectomy, hepatic resection

## Abstract

Major hepatectomy (MH) has traditionally been associated with higher R0 rates in colorectal liver metastases (CRLM), but at the cost of increased morbidity. Parenchymal-sparing hepatectomy (PSH) has emerged as an alternative approach aimed at reducing perioperative complications while preserving functional liver parenchyma without compromising oncological outcomes. We retrospectively analyzed 248 consecutive patients undergoing liver resection for CRLM between 2016 and 2025, classified as PSH (*n* = 215, 86.7%) or MH (*n* = 33, 13.3%). MH was performed more frequently in patients with greater tumor burden, including larger lesions, more numerous metastases, and bilobar disease (all *p* < 0.001). PSH was associated with shorter hospital stay, fewer postoperative complications, and lower 30-day readmission rate. In multivariable Cox analyses, surgical strategy was not associated with recurrence-free survival or overall survival, which were primarily driven by tumor burden. Among patients who developed liver recurrence, repeat hepatectomy was more often feasible after PSH than MH (*p* = 0.026), emphasizing the long-term value of preserving functional parenchyma. Overall, PSH was associated with lower postoperative morbidity, enabling earlier recovery, while facilitating future liver resections when needed in this chronically evolving disease.

## Simple Summary

The liver is the most frequent site of metastases from colorectal cancer, and surgery remains the only potential curative treatment when the disease is resectable. Traditionally, major liver resections were used to ensure complete removal of the metastases, but they are associated with a higher risk of postoperative complications. Over time, parenchymal-sparing techniques have become increasingly adopted, aiming to remove all metastases while preserving as much healthy liver as possible. In this single-center study, we compared outcomes of patients treated with major hepatectomy versus parenchymal-sparing resections. We found that sparing techniques were associated with fewer complications and similar long-term survival. Importantly, patients who preserved more liver tissue were more likely to undergo repeat hepatectomy if the cancer recurred in the liver, an important consideration given the chronic nature of this disease. These findings support adoption of parenchymal-sparing approaches whenever technically and oncologically feasible.

## 1. Introduction

Major hepatectomy (MH) has long been considered the standard approach for colorectal liver metastases (CRLM). However, this approach is burdened by high postoperative complication rates, the most feared of which is post-hepatectomy liver failure [1,2,3]. In addition, especially with the increasing adoption of a multidisciplinary management, CRLM have become a chronic disease for many patients, who may require repeat hepatic resections, which can be limited after major hepatectomy [4].

For these reasons, in recent years parenchymal-sparing hepatectomy (PSH) has progressively gained attention. Evidence shows that a negative margin > 1 mm does not increase recurrence risk in CRLM, and that R1vasc (R1 resection with detachment of CRLM from major intrahepatic vessels) resections allow preservation of parenchyma without compromising oncological outcomes [5,6].

Growing evidence on PSH has shown that this strategy provides oncological outcomes comparable to MH, while reducing postoperative morbidity, and may expand resectability criteria even in cases of liver recurrence [7,8,9].

The aim of this study was to analyze our cohort over the past decade to identify whether, and under which circumstances, PSH conferred advantages over MH regarding postoperative and oncological outcomes.

## 2. Materials and Methods

We retrospectively analyzed clinical data from a prospectively maintained database of patients who underwent hepatic resections for CRLM between January 2016 and March 2025 at the Morgagni-Pierantoni Hospital in Forlì.

All consecutive patients who underwent hepatic resection for CRLM with curative intent were included in the analysis. Intraoperative ultrasound (IOUS) was routinely performed to assess the number and location of metastases and to guide resection. All resections were carried out with R0 or R1-vascular intent. Patients treated exclusively with laparoscopic radiofrequency ablation (RFA) were excluded, whereas those who underwent RFA in addition to hepatic resection were included. The decision to ablate or resect each lesion was made at the surgeon’s discretion, based on lesion size and location; in our practice, RFA was considered for lesions up to 2 cm in maximum diameter. Similarly, the decision to perform a Pringle maneuver was left to the surgeon’s discretion, based on the difficulty of resection and the presence of underlying chronic liver disease. When applied, intermittent clamping–unclamping followed one of two standardized cycles (7 min clamp + 3 min release or 15 min clamp + 5 min release). In minimally invasive surgery, an intracorporeal Pringle maneuver was performed using a Foley catheter tightened around the hepatic pedicle under direct vision.

Perioperative chemotherapy or chemoradiotherapy was administered according to multidisciplinary discussion and current guidelines. For synchronous liver metastases, the timing of liver resection (liver-first, primary-first, or simultaneous resection) was determined during multidisciplinary oncological meetings. In patients undergoing simultaneous resection, bowel and liver procedures were performed by separate colorectal and hepatobiliary specialized teams, respectively. For each patient, demographic data, comorbidities (ASA score, Charlson Comorbidity Index, modified 5-item frailty index), history of previous abdominal surgery, number and size of metastases, bilobar involvement, synchronous/metachronous presentation, associated ablative procedures, operative time (intended as total operative time, not just resection time) and surgical approach (open vs. minimally invasive approach) were collected. Postoperative complications were graded according to the Clavien-Dindo classification, with major complications defined as grade ≥ III [10]. Liver surgical-site infection (liver-SSI) was defined as an infected collection at the resection site, diagnosed by radiological imaging and requiring either antibiotic therapy or radiological drainage. Since there is no standardized definition of postoperative ascites, we adopted the definition used by Yoshikawa et al., considering a postoperative drainage output ≥ 500 mL on postoperative day 3 or later [11]. Post-hepatectomy liver failure (PHLF) was classified according to ISGLS definition [2].

Resections involving three or more contiguous segments were classified as MH, whereas all other resections were categorized as parenchymal-sparing hepatectomy [12]. The MH included both single-stage and two-stage hepatectomies. For patients undergoing a two-stage procedure, only the second operation, corresponding to the actual MH, was considered for the analysis of postoperative outcomes. However, for survival analyses, postoperative complications were assessed cumulatively across both stages of the two-stage hepatectomy, as the overall postoperative course was deemed more representative of the patient’s recovery.

Recurrence-free survival (RFS) was defined as the interval between surgery and radiologically documented recurrence. Overall survival (OS) was calculated from surgery to death or last follow-up.

Categorical variables are reported as frequencies and percentages, whereas continuous variables are reported as mean ± standard deviation (SD) or median with interquartile range (IQR), as appropriate. Categorical variables were compared with the chi-square test or Fisher’s exact test, and continuous variables with Mann–Whitney U test or Kruskal–Wallis test, as appropriate.

Univariable and multivariable logistic regression analyses were performed to identify factors associated with MH, and postoperative outcomes. Variables with a *p*-value < 0.10 in univariable analysis were included in the multivariable model.

Univariable and multivariable Cox proportional hazards regression models were used to assess predictors of RFS and OS. Variables with a *p* < 0.10 in univariable analysis entered the multivariable Cox models. A *p*-value < 0.05 was considered statistically significant.

Data analysis was performed using Jamovi software (version 2.6.44).

## 3. Results

A total of 248 patients were included: 215 (86.7%) underwent PSH and 33 (13.3%) underwent MH. Among the MH group, 14 patients underwent two-stage hepatectomy (42.4%) and 19 underwent single-stage hepatectomy (57.6%). The demographic and tumor characteristics are shown in Table 1 and Table 2, respectively. MH was performed in patients with a higher oncological burden, including larger lesions, a greater number of metastases, and bilobar involvement. This likely explains the higher rate of patients receiving neoadjuvant chemotherapy (51.2% in PSH vs. 84.8% in MH, *p* <0.001). Age, sex, ASA, and CCI did not differ significantly between groups (Table 1).

In the multivariable logistic regression performed with MH as the dependent variable (Table S1), the presence of a lesion > 5 cm was the strongest factor associated with the choice of MH (OR 8.63, 95% CI 3.33–22.39; *p* < 0.001).

The intraoperative characteristics of the patients are shown in Table 3. MIS was more common in PSH, although this difference did not reach statistical significance. MH was associated with a significantly longer operative time. The use of associated RFA was similar across groups. Although synchronous disease was more common in the MH cohort (72.7% vs. 51.5%, *p* = 0.022), simultaneous resection of the primary tumor was more frequent in the PSH group (23.3% vs. 6.1%, *p* = 0.003).

### 3.1. Postoperative Complications (Table 4)

Overall postoperative morbidity was significantly higher after MH compared with PSH (69.7% vs. 37.7%, *p* < 0.001). However, rates of major complications (Clavien-Dindo ≥ III) did not differ significantly. MH was associated with higher rates of postoperative ascites (18.20% vs. 0.50%, *p* < 0.001), PHLF (12.1% vs. 0.5%, *p* = 0.001), liver-SSI (24.2% vs. 11.2%, *p* = 0.037), and bile leak (24.2% vs. 3.7%, *p* < 0.001). Length of hospital stay and 30-day readmission were higher in the MH cohort (*p* < 0.001 and *p* = 0.003, respectively).

**Table 4:** Postoperative characteristics in patients who underwent PSH or MH for CRLM. PSH, parenchymal-sparing hepatectomy; MH, major hepatectomy; IQR, interquartile range; AKI, acute kidney injury; PHLF, post-hepatectomy liver failure.

	PSH (*n* = 215)	PSH (% = 86.7%)	MH (*n* = 33)	MH (% = 13.3%)	*p*-Value
**Hospital stay, median (days) [IQR]**	7 [6–10]		9 [7–16]		<0.001
**Overall complications**	81	37.7%	23	69.7%	<0.001
**Major complications (Clavien ≥ 3)**	27	12.6%	6	18.2%	0.376
**Ascites**	1	0.5%	6	18.2%	<0.001
**AKI**	5	2.3%	1	3.0%	0.579
**PHLF**	1	0.5%	4	12.1%	0.001
**Liver-SSI**	24	11.2%	8	24.2%	0.037
**Bile leak**	8	3.7%	8	24.2%	<0.001
**Cardiovascular complications**	3	1.4%	1	3.0%	0.437
**Pulmonary complications**	21	9.8%	5	15.2%	0.360
**Other complications**	22	10.4%	4	12.5%	0.758
**Reintervention**	3	1.4%	0	0.0%	1.000
**30-day readmission**	10	4.7%	7	21.2%	0.003
**90-day mortality**	1	0.5%	0	0.0%	1.000
**R1-resection**	32	15.8%	5	15.6%	0.993

In the multivariable analysis (Table 5), MH remained an independent predictor of postoperative complications (OR 3.99, 95% CI 1.55–10.32; *p* = 0.004), but not of major complications or liver-SSI (Table 6 and Table S2, respectively). Simultaneous resection for synchronous disease independently increased the risk of postoperative complications (OR 2.61, 95% CI 1.08–6.30; *p* = 0.033). MIS strongly reduced postoperative morbidity (OR 0.26, 95% CI 0.15–0.46; *p* < 0.001). Longer operative time was modestly associated with postoperative complications, major complications, and liver-SSI.

### 3.2. Oncological Outcomes (Table 7)

The median follow-up for the overall population was 43.3 months (95% CI, 35.6–52.7). MH appeared to have a higher rate of postoperative recurrence (60.3% in PSH vs. 84.4% in MH, *p* = 0.008), with no differences in recurrence sites (*p* = 0.415). This may be explained by the higher tumor burden of patients who underwent MH, rather than the surgical strategy itself. No difference was found in the proportion of patients who experienced liver-only recurrence. Interestingly, among patients with liver recurrence, the rate of those undergoing repeat hepatectomy was higher in the PSH group (*p* = 0.026). No difference in terms of patients receiving adjuvant chemotherapy was found.

**Table 7:** Oncological outcomes in patients who underwent PSH or MH for CRLM. PSH, parenchymal-sparing hepatectomy; MH, major hepatectomy.

	PSH (*n* = 215)	PSH (% = 86.7%)	MH (*n* = 33)	MH (% = 13.3%)	*p*-Value
**Adjuvant chemotherapy**	96	52.5%	15	51.7%	0.941
**Recurrence**	126	60.3%	27	84.4%	0.008
**Liver recurrence**	55/126	43.7%	10/27	37.0%	0.415
**Extrahepatic recurrence**	40/126	31.7%	7/27	25.9%	
**Hepatic + extrahepatic recurrence**	31/126	24.6%	10/27	37.0%	
**Hepatectomy for liver recurrence**	19	15.1%	0	0.0%	0.026

### 3.3. Recurrence-Free Survival

RFS appeared worse in the MH group in univariable analysis; however, this association did not persist after adjustment. In the multivariable Cox model (Table S3), surgical strategy (PSH vs. MH) did not correlate with recurrence (HR 1.39, 95% CI 0.85–2.27; *p* = 0.187). RFS was instead influenced by tumor-related factors such as more than 5 metastases (HR 2.11, 95% CI 1.25–3.55; *p* = 0.005), and lesions > 5 cm (HR 2.06, 95% CI 1.30–3.25; *p* = 0.002). Postoperative complications were also associated with worse RFS (HR 1.53, 95% CI 1.09–2.16, *p* =0.015).

### 3.4. Overall Survival

The median OS was 57.1 months (95% CI 46.6–73.6) in the PSH group, and 32.3 months (95% CI 26.2–NA) in the MH group. In the univariable Cox analysis, MH was associated with worse OS (HR 1.99, 95% CI 1.16–3.43; *p* = 0.013). However, similar to RFS, OS did not differ significantly between PSH and MH on multivariable analysis (HR 1.35, 95% CI 0.70–2.60; *p* = 0.377) (Table 8). Survival outcomes were mainly determined by oncologic burden rather than by the extent of hepatic resection.

## 4. Discussion

In recent years, PSH has emerged as an alternative to MH for the treatment of CRLM with the aim of preserving functional liver parenchyma without affecting oncological outcomes [13,14,15,16].

In our single-center cohort, MH was mainly reserved for patients with a higher hepatic tumor burden compared with PSH. Particularly, the presence of a lesion > 5 cm emerged as the strongest factor associated with choosing MH, whereas demographic characteristics of patients, such as age or comorbidity (including ASA, CCI, and mFI-5), did not seem to influence the surgical strategy.

Given the more complex disease patterns, MH was associated with a higher rate of overall postoperative complications (but not major complications), particularly ascites, perihepatic collection, bile leakage, and, unsurprisingly, PHLF (all classified as grade A or B according to ISGLS [2]). This translated into longer hospital stay and a higher 30-day readmission rate, although 90-day mortality did not differ between groups. On multivariable analysis, MH remained an independent predictor of overall complications, but not of major complications or liver-SSI. Minimally invasive surgery was a protective factor for overall complications. Notably, major complications were independently associated with age ≥ 75 at the time of surgery.

R0/R1 resection rates were similar between PSH and MH (*p* = 0.993), consistent with current evidence supporting the oncological adequacy of PSH [16,17,18]. Although recurrence was more frequent after MH than PSH in univariable analysis, this association disappeared after adjustment for tumor burden. In the multivariable Cox model, RFS was mainly driven by tumor-related factors, such as lesions size > 5 cm and more than five metastases, rather than by the extent of resection. RFS was also associated with overall postoperative complications, possibly through delayed recovery, impaired liver regeneration, or postponed initiation of adjuvant chemotherapy [19,20,21]. In this regard, PSH might be a protective factor reducing postoperative complications and allowing earlier access to adjuvant chemotherapy. However, in our cohort, the rate of patients receiving adjuvant chemotherapy did not differ between the two groups.

Similarly, in the multivariable Cox model, surgical strategy did not influence OS. Once again, OS was mainly determined by tumor burden and patient condition, including frailty and comorbidity. Indeed, an mFI-5 ≥ 2 was independently associated with worse OS. This is likely to reflect the greater vulnerability of frail patients, which is often linked to advanced age; however, in our cohort, age > 75 was not independently associated with OS. Notably, mFI-5 was not associated with RFS, suggesting that the poorer OS observed in these patients may be driven by non-cancer-related factors. Our findings regarding RFS and OS being mainly determined by tumor burden characteristics were consistent with previous studies [22,23,24].

Taken together, these findings suggest that, in our cohort, the primary advantage of PSH lies in the reduction of overall postoperative morbidity, not in oncological superiority.

Importantly, although recurrence patterns did not differ between MH and PSH, in our cohort, patients who underwent PSH were more likely to receive a repeat hepatectomy for liver recurrence, emphasizing the long-term value of preserving healthy parenchyma. While the higher rate of repeat hepatectomy observed in the PSH group was likely influenced by differences in tumor burden, it is also plausible that parenchymal preservation facilitated repeat hepatectomy at recurrence. Previous studies reported similar findings and showed that repeat hepatectomy improves OS in patients with recurrent CRLM [25,26,27]. Consistent with these studies, the main benefit of PSH appears to be the preservation of liver parenchyma, which allows repeat resection rather than alternative treatments such as ablation or systemic therapy. When considering liver-only recurrence, tumor characteristics did not differ between patients who previously underwent PSH or MH [25], suggesting that the recurrence pattern was not affected by resection strategy. The choice of operative strategy becomes particularly relevant in patients with synchronous CRLM. In our cohort, MH was more frequently performed in patients with synchronous disease, yet simultaneous colorectal-hepatic resection was significantly less common among MH patients. Multivariable analysis confirmed both simultaneous resection and MH as independent predictors of postoperative complications. Moreover, simultaneous procedures typically involve longer operative times, which in our cohort was associated with an increased risk of complications. Given these data, consistent with previous literature [28,29], MH should be performed with caution during simultaneous resections for patients with synchronous disease in whom adjuvant treatments might be particularly important. In this setting, minimizing postoperative morbidity is crucial, as any complication may delay adjuvant chemotherapy and compromise survival. Notably, in our cohort, PSH was associated with lower morbidity despite being the most common strategy in simultaneous cases.

Overall, our results add to the growing evidence that PSH is a feasible and safe option for CRLM when technically and oncologically appropriate. Its main advantage appears to be the reduction in postoperative morbidity, resulting in shorter hospital stay, and the preservation of functional liver parenchyma for potential repeat resections. This aspect may be particularly valuable in patients undergoing simultaneous colorectal and liver surgery.

Although we did not evaluate a learning curve, this was largely because the core hepatobiliary team had already completed its learning curve before the study period and the multidisciplinary leadership remained stable over time.

Surgeons aiming to adopt PSH should be aware that specific technical skills and resources are required, particularly when extending parenchymal preservation to complex disease patterns. Intraoperative ultrasound (IOUS) is essential for liver “navigation,” enabling real-time lesion detection, definition of tumor–vessel relationships, and guidance of transection planes—especially when pursuing vascular-adjacent resections, including R1-vascular strategies in selected cases. Routine IOUS also supports a highly reliable intraoperative staging, allowing refinement of the operative plan based on real-time assessment of lesion number and distribution. Advances in parenchymal transection (e.g., ultrasonic devices and advanced bipolar instruments) have facilitated more controlled dissection, particularly in steatotic or fibrotic livers and around Glissonian pedicles and hepatic veins. Proficiency with ablative techniques (RFA and microwave ablation) can further support combined strategies aimed at maximizing parenchymal preservation. Finally, implementing PSH in routine practice often benefits from capability in both open and minimally invasive approaches, tailored to disease distribution and technical complexity [30].

The main limitations of our study include its retrospective design and the relatively small number of MH procedures. Differences in tumor burden between PSH and MH groups represent a major limitation of this study and may have influenced postoperative and oncological outcomes, particularly recurrence and repeat hepatectomy rates. To adjust for potential cofounders, multivariable analysis were performed for postoperative complications, RFS, and OS, when supported by an adequate number of events. Additionally, the MH group included patients undergoing two-stage hepatectomy, which may have contributed to higher morbidity in this subgroup. Propensity score methods were not applied, which may have further reduced selection bias, particularly given the imbalance between groups.

## 5. Conclusions

When technically and oncologically feasible, parenchymal sparing hepatectomy should be considered the preferred strategy for CRLM, as it is associated with fewer postoperative complications while no differences in OS and RFS were found compared to MH. This advantage becomes particularly relevant in the setting of synchronous disease, where minimizing postoperative morbidity is crucial to safely perform simultaneous colorectal and liver resections and to avoid delays in adjuvant therapy. Moreover, by preserving functional liver parenchyma, PSH may increase the chances of repeated hepatectomy in patients who developed intrahepatic recurrence.

## Figures and Tables

**Table 1:** Demographic characteristics in patients who underwent PSH or MH for CRLM. PSH, parenchymal-sparing hepatectomy; MH, major hepatectomy; IQR, interquartile range; BMI, body mass index; ASA, American Society of Anesthesiologists; CCI, Charlson comorbidity Index; mFI-5, modified 5-item frailty index.

	PSH (*n* = 215)	PSH (% = 86.7%)	MH (*n* = 33)	MH (% = 13.3%)	*p*-Value
**Sex (male)**	129	60.0%	25	75.8%	0.082
**Age, median [IQR]**	68.2 [57.5–75.9]		63.7 [57.6–71.5]		0.059
**Age > 75**	63	29.3%	7	21.2%	0.336
**BMI, median [IQR]**	25.9 [23.4–28.6]		24.2 [22.9–27.6]		0.217
**ASA ≥ 3**	103	47.9%	15	45.5%	0.793
**CCI ≥ 9**	102	47.4%	21	63.6%	0.083
**mFI-5 ≥ 2**	179	83.3%	27	81.8%	0.838

**Table 2:** Tumor characteristics in patients who underwent PSH or MH for CRLM. PSH, parenchymal-sparing hepatectomy; MH, major hepatectomy.

	PSH (*n* = 215)	PSH (% = 86.7%)	MH (*n* = 33)	MH (% = 13.3%)	*p*-Value
**Rectal primary**	54	25.1%	11	33.3%	0.318
**Synchronous metastases**	110	51.2%	24	72.7%	0.022
**>5 lesions**	34	15.8%	15	45.5%	<0.001
**Bilobar metastases**	50	23.3%	19	57.6%	<0.001
**Neoadjuvant chemotherapy**	110	51.2%	28	84.8%	<0.001
**Lesion ≥ 5 cm**	20	9.3%	13	39.4%	<0.001

**Table 3:** Intraoperative characteristics in patients who underwent PSH or MH for CRLM. PSH, parenchymal-sparing hepatectomy; MH, major hepatectomy; IQR, interquartile range; RFA, radiofrequency ablation; MIS, minimally invasive surgery.

	PSH (*n* = 215)	PSH (% = 86.7%)	MH (*n* = 33)	MH (% = 13.3%)	*p*-Value
**Simultaneous resection of primary tumor**	63	29.3%	2	6.1%	0.003
**Operative time, median (min) [IQR]**	330 [250–420]		400 [347–430]		0.001
**RFA**	27	12.6%	5	16.1%	0.570
**MIS**	123	57.2%	13	39.4%	0.056
**Conversion to laparotomy**	17/123	13.8%	4/13	30.8%	0.108
**Pringle time, median (min) [IQR]**	42 [25–70]		48.5 [35–62.3]		0.580
**Intraoperative complications**	8	3.7%	1	3.0%	0.840
**Intraoperative transfusions**	15	7.0%	5	15.2%	0.159

**Table 5:** Multivariable logistic regression for predictors of postoperative complications. OR, odds ratio; CI, confidence interval; CCI, Charlson comorbidity Index; ASA, American Society of Anesthesiologists; RFA, radiofrequency ablation; MH, major hepatectomy; MIS, minimally invasive surgery.

Overall Complications (*n* = 104)				
	Univariable OR (95% CI)	*p*-Value	Multivariable OR (95% CI)	*p*-Value
**Age ≥ 75**	1.58 [0.91–2.76]	0.108		
**Sex (male)**	0.73 [0.43–1.24]	0.242		
**CCI ≥ 9**	0.96 [0.58–1.59]	0.881		
**ASA III–IV**	0.69 [0.42–1.16]	0.158		
**Previous abdominal surgery**	0.76 [0.41–1.40]	0.385		
**Metachronous metastases**	0.59 [0.35–0.99]	0.045	1.597 [0.723–3528]	1.597
**>5 lesions**	1.95 [1.04–3.66]	0.039	0.87 [0.34–2.26]	0.873
**Bilobar lesions**	1.93 [1.10–3.39]	0.021	1.27 [1.53–3.02]	0.595
**Dimension ≥ 5 cm**	1.18 [0.57–2.47]	0.660		
**RFA**	1.43 [0.68–3.02]	0.345		
**Simultaneous resection**	2.29 [1.29–4.07]	0.005	2.61 [1.08–6.30]	0.033
**MH**	3.81 [1.72–8.40]	<0.001	3.99 [1.55–10.32]	0.004
**MIS**	0.25 [0.15–0.43]	<0.001	0.26 [0.15–0.46]	<0.001
**Operative time**	1.010 [1.002–1.007]	<0.001	1.003 [1.001–1.006]	0.02
**Pringle time**	1.00 [0.99–1.01]	0.956		

**Table 6:** Multivariable logistic regression for predictors of major complications (Clavien-Dindo > 3). OR, odds ratio; CI, confidence interval; CCI, Charlson comorbidity Index; ASA, American Society of Anesthesiologists; RFA, radiofrequency ablation; MH, major hepatectomy; MIS, minimally invasive surgery.

Major Complications (*n* = 33)				
	Univariable OR (95% CI)	*p*-Value	Multivariable OR (95% CI)	*p*-Value
**Age ≥ 75**	2.09 [0.98–4.45]	0.055	2.25 [1.10–4.88]	0.040
**Sex (male)**	0.80 [0.37–1.72]	0.562		
**CCI ≥ 9**	0.62 [0.29–1.31]	0.211		
**ASA III-IV**	0.91 [0.43–1.89]	0.793		
**Previous abdominal surgery**	0.58 [0.26–1.30]	0.183		
**Metachronous metastases**	0.85 [0.40–1.78]	0.661		
**>5 lesions**	0.69 [0.25–1.90]	0.477		
**Bilobar lesions**	0.97 [0.43–2.20]	0.940		
**Dimension ≥ 5 cm**	0.62 [0.18–2.15]	0.448		
**RFA**	1.62 [0.61–4.28]	0.335		
**Simultaneous resection**	1.75 [0.81–3.79]	0.158		
**MH**	1.55 [0.59–4.09]	0.379		
**MIS**	0.75 [0.36–1.55]	0.432		
**Operative time**	1.004 [1.001–1.006]	0.009	1.004 [1.001–1.006]	0.006
**Pringle time**	1.01 [0.99–1.01]	0.124		

**Table 8:** Cox multivariable regression analysis for overall survival. HR, hazard ratio; CCI, Charlson comorbidity Index; ASA, American society of anesthesiologists; mFI-5, modified 5-item frailty index; MH, major hepatectomy; MIS, minimally invasive surgery. AKI, acute kidney injury, PHLF, post-hepatectomy liver failure; SSI, surgical site infection.

	Overall Survival			
	Univariable HR (95% CI)	*p*-Value	Multivariable HR (95% CI)	*p*-Value
**Age > 75**	1.47 [0.96–2.25]	*p* = 0.074		
**Sex (male)**	0.79 [0.51–1.21]	*p* = 0.278		
**CCI ≥ 9**	1.33 [0.87–2.02]	*p* = 0.191		
**ASA ≥ 3**	1.45 [0.95–2.20]	*p* = 0.086		
**mFI-5 ≥ 2**	1.83 [1.11–3.01]	*p* = 0.017	2.41 [1.33–4.38]	*p* = 0.004
**Rectal primary**	1.18 [0.74–1.88]	*p* = 0.489		
**Metachronous metastases**	0.77 [0.51–1.17]	*p* = 0.221		
**>5 lesions**	2.01 [1.21–3.34]	*p* = 0.007	2.89 [1.64–5.10]	*p* < 0.001
**Bilobar lesions**	1.31 [0.83–2.08]	*p* = 0.247		
**Lesion ≥ 5 cm**	2.14 [1.29–3.54]	*p* = 0.003	3.12 [1.69–5.76]	*p* < 0.001
**MH**	2.03 [1.18–3.50]	*p* = 0.010	1.35 [0.70–2.60]	*p* = 0.377
**MIS**	0.74 [0.49–1.13]	*p* = 0.165		
**Ascites**	8.64 [3.37–22.18]	*p* < 0.001	0.67 [0.15–3.02]	*p* = 0.598
**AKI**	4.84 [1.95–12.03]	*p* = 0.001	1.60 [0.37–6.98]	*p* = 0.532
**PHLF**	0.00 [0.00–∞]	*p* = 0.996		
**Liver-SSI**	1.20 [0.63–2.25]	*p* = 0.580		
**Biliary Leak**	1.10 [0.51–2.38]	*p* = 0.811		
**Cardiovascular complications**	21.64 [6.49–72.21]	*p* < 0.001)	6.13 [0.63–59.88]	*p* = 0.119
**Pulmonary complications**	2.54 [1.43–4.52]	*p* = 0.001	2.86 [1.38–5.93]	*p* = 0.005
**Reintervention**	0.59 [0.08–4.23]	*p* = 0.598		
**30-day readmission**	1.01 [0.46–2.19]	*p* = 0.989		
**Overall complications**	1.68 [1.11–2.55]	*p* = 0.015	1.36 [0.83–2.24]	*p* = 0.222
**Major complications (Clavien ≥ 3)**	1.49 [0.85–2.60]	*p* = 0.161		

## Data Availability

In accordance with current privacy legislation, the database generated and analyzed during this study cannot be publicly shared.

## Supplementary Materials

The following supporting information can be downloaded at: https://www.mdpi.com/article/10.3390/curroncol33010046/s1. Table S1: Multivariable logistic regression for predictors for major hepatectomy. Table S2: Multivariable logistic regression for predictors of perihepatic collection. Table S3: Cox multivariable regression analysis for recurrence free survival.

## Abbreviations

The following abbreviations are used in this manuscript:

MH	Major hepatectomy
CRLM	Colorectal liver metastases
PSH	Parenchyma-sparing hepatectomy
IOUS	Intraoperative ultrasound
RFA	Radiofrequency ablation
ASA	American society of anesthesiologists
RFS	Recurrence free survival
OS	Overall survival
SD	Standard deviation
IQR	Interquartile range
CCI	Charlson comorbidity index
BMI	Body mass index
mFI-5	Modified 5-item frailty index
OR	Odds ratio
CI	Confidence interval
PHLF	Post-hepatectomy liver failure
