# Supplementary material for: Parenchymal-Sparing Strategy in Colorectal Liver Metastases: A Single-Center Experience
Source: Curr Oncol. 2026 Jan 15;33(1):46. doi: 10.3390/curroncol33010046 (PMC12840290; doi:10.3390/curroncol33010046)
Supplement: Supplementary file 1 [file curroncol-33-00046-s001.zip › curroncol-4063239-supplementary.pdf]

Table S1. Multivariable logistic regression for predictors for major hepatectomy. OR, odds ratio; CI, confidence interval; BMI, body mass index; CCI, Charlson comorbidity Index; ASA, American Society of Anesthesiologists; mFI-5, modified 5-item frailty index.

Major hepatectomy (*n* = 33)				
	**Univariable OR (95% CI)**	**p-value**	**Multivariable OR (95% CI)**	**p-value**
**Age ≥75**	0.65 [0.27-1.57]	0.339		
**Sex (male)**	0.48 [0.20-1.14]	0.087		
**BMI**	0.97 [0.89-1.05]	0.452		
**CCI ≥9**	1.94 [0.91-4.14]	0.087		
**ASA III-IV**	0.61 [0.43-1.89]	0.793		
**mFI-5 ≥2**	0.91 [0.35-2.35]	0.838		
**Previous abdominal surgery**	3.03 [0.89-10.35]	0.077		
**Metachronous metastases**	0.39 [0.17-0.88]	0.024	0.60 [0.23-1.51]	0.279
**>5 lesions**	4.43 [2.04-9.65]	<.001	3.15 [0.94-10.58]	0.063
**Bilobar lesions**	4.48 [2.10-9.57]	<.001	2.03 [0.65-6.33]	0.220
**Lesion ≥5 cm**	6.34 [2.75-14.63]	<.001	8.63 [3.33-22.39]	<.001

Table S2. Multivariable logistic regression for predictors of perihepatic collection. SSI, surgical site infection; OR, odds ratio; CI, confidence interval; CCI, Charlson comorbidity Index; ASA, American Society of Anesthesiologists; RFA, radiofrequency ablation; MH, major hepatectomy; MIS, minimally invasive surgery.

Liver-SSI (*n* = 32)				
	**Univariable OR (95% CI)**	**p-value**	**Multivariable OR (95% CI)**	**p-value**
**Age ≥75**	0.99 [0.44-2.27]	0.989		
**Sex (male)**	1.32 [0.62-2.80]	0.466		
**CCI ≥9**	1.36 [0.64-2.87]	0.421		
**ASA III-IV**	0.84 [0.40-1.77]	0.642		
**Previous abdominal surgery**	0.97 [0.39-2.38]	0.941		
**Metachronous metastases**	0.58 [0.26-1.25]	0.162		
**>5 lesions**	1.42 [0.60-3.39]	0.427		
**Bilobar lesions**	1.67 [0.77-3.64]	0.194		
**Dimension ≥5 cm**	0.64 [0.18-2.24]	0.641		
**RFA**	3.25 [1.34-7.86]	0.009	2.45 [0.95-6.32]	0.064
**Simultaneous resection**	1.57 [0.71-3.47]	0.263		
**MH**	2.55 [1.03-6.28]	0.042	1.85 [0.67-5.06]	0.233
**MIS**	0.69 [0.33-1.46]	0.334		
**Operative time**	1.004 [1.002-1.007]	0.002	1.0030 [1.0004-1.0065]	0.026
**Pringle time**	1.00 [0.99-1.01]	0.611		
**Biliary leak**	3.45 [1.11-10.69]	0.032	2.59 [0.74-9.00]	0.135
**Ascites**	1.13 [0.13-9.70]	0.912		

Table S3. Cox multivariable regression analysis for recurrence free survival. HR, hazard ratio; CCI, Charlson comorbidity index; ASA, American society of anesthesiologists; mFI-5, modified 5-item frailty index; nCHT, neoadjuvant chemotherapy; MH, major hepatectomy; RFA, radiofrequency ablation; MIS, minimally invasive surgery.

Recurrence free survival				
	**Univariable HR (95% CI)**	**p-value**	**Multivariable HR (95% CI)**	**p-value**
**Age >75**	0.86 [0.60-1.22]	p=0.393		
**CCI ≥9**	0.96 [0.70-1.32]	p=0.823		
**ASA≥3**	1.11 [0.81-1.53])	p=0.510		
**mFI-5≥2**	0.96 [0.62-1.48],	p=0.853		
**Rectal primary**	0.92 0.64-1.33]	p=0.648		
**Metachronous metastases**	0.77 [0.56-1.06]	p=0.114		
**>5 lesions**	2.14 [1.48-3.10]	p<0.001	2.11 [1.25-3.55]	p=0.005
**Bilobar lesions**	1.66 [1.19-2.33]	p=0.003	0.74 [0.45-1.22]	p=0.241
**nCHT**	1.76 [1.26-2.45]	p=0.001	1.41 [0.96-2.06]	p=0.080
**Lesion ≥5 cm**	2.08 [1.37-3.14]	p=0.001	2.06 [1.30-3.25]	p=0.002
**Simultaneous resection**	0.98 [0.68-1.40]	p=0.907		
**MH**	2.54 [1.65-3.91]	p<0.001	1.39 [0.85-2.27]	p=0.187
**RFA**	1.57 [1.01-2.45]	p=0.046	1.41 [0.86-2.32]	p=0.174
**MIS**	0.63 [0.46-0.87]	p=0.005	0.71 [0.50-1.00]	p=0.052
**Overall complications**	1.69 [1.23-2.32]	p=0.001	1.53 [1.09-2.16]	p=0.015
**Major complications (Clavien ≥3)**	1.41 [0.92-2.17])	p=0.115		
